# The structure of mollusc larval shells formed in the presence of the chitin synthase inhibitor Nikkomycin Z

**DOI:** 10.1186/1472-6807-7-71

**Published:** 2007-11-06

**Authors:** Veronika Schönitzer, Ingrid M Weiss

**Affiliations:** 1 Lehrstuhl Biochemie I, Universität Regensburg, Universitätsstr. 31, 93053 Regensburg, Germany; 2 INM – Leibniz-Institut für Neue Materialien gGmbH, Campus D2 2, 66123 Saarbrücken, Germany

## Abstract

**Background:**

Chitin self-assembly provides a dynamic extracellular biomineralization interface. The insoluble matrix of larval shells of the marine bivalve mollusc *Mytilus galloprovincialis *consists of chitinous material that is distributed and structured in relation to characteristic shell features. Mollusc shell chitin is synthesized via a complex transmembrane chitin synthase with an intracellular myosin motor domain.

**Results:**

Enzymatic mollusc chitin synthesis was investigated *in vivo *by using the small-molecule drug NikkomycinZ, a structural analogue to the sugar donor substrate UDP-N-acetyl-D-glucosamine (UDP-GlcNAc). The impact on mollusc shell formation was analyzed by binocular microscopy, polarized light video microscopy *in vivo*, and scanning electron microscopy data obtained from shell material formed in the presence of NikkomycinZ. The partial inhibition of chitin synthesis *in vivo *during larval development by NikkomycinZ (5 μM – 10 μM) dramatically alters the structure and thus the functionality of the larval shell at various growth fronts, such as the bivalve hinge and the shell's edges.

**Conclusion:**

Provided that NikkomycinZ mainly affects chitin synthesis in molluscs, the presented data suggest that the mollusc chitin synthase fulfils an important enzymatic role in the coordinated formation of larval bivalve shells. It can be speculated that chitin synthesis bears the potential to contribute via signal transduction pathways to the implementation of hierarchical patterns into chitin mineral-composites such as prismatic, nacre, and crossed-lamellar shell types.

## Background

Molluscs were among the first organisms on earth that were able to produce highly organized calcium carbonate composite materials with unique structural features and remarkable materials properties [1,2]. Even today, the mollusc shell surprises us with new concepts for understanding biomineralization processes [3].

Chitin, a linear homopolymer consisting of β-(1–4)-linked N-acetyl-D-glucosamine subunits, plays an important role in mollusc shell formation. The presence of chitin in mollusc shell matrices is well documented in the literature [4-6]. Recently, it has been demonstrated that chitin fulfils various structural tasks in the formation of larval shells of the bivalve mollusc *Mytilus galloprovincialis *[7]. In the adult stage, the fibres of chitin and certain crystallographic axes of aragonite are aligned in mollusc nacre [8,9]. Based on a cryo-TEM study, Levi-Kalisman and colleagues suggested that chitin is the ordered component of the decalcified nacre matrix, whereas a silk-like protein gel environs the chitin sheets [10]. Mineralizing proteins are either attached to the core sheets or distributed within the silk like protein gel. Thus, the chitin and the silk together form a regular lamellar structure. Subsequently, certain mollusc shell protein fractions induce aragonite formation within this lamellar β-chitin and silk framework [11]. The involvement of a transient amorphous mineral precursor phase in the formation of aragonite biominerals is currently discussed based on the presence of such a phase in calcite forming sea urchins and aragonite forming mollusc larvae [3,12-14]. These observations raised new questions regarding the role of structural biopolymers such as chitin in the formation of shell microtextures [15]. As recently discovered, the calcitic prismatic layer of the bivalve mollusc *Atrina rigida *(Pteriomorphia, Pinnidae) is the result of a structural interplay between chitin and the mineral phases [16].

Chitin synthases are transmembrane glycosyltransferases that are responsible for the enzymatic synthesis of chitin [17]. The representatives of these enzymes in molluscs contain a N-terminal myosin motor head domain [18]. For many years, the chitin synthases have been studied mainly in fungi [19-23]. Chitin synthases are localized either in the plasma membrane or in so-called chitosomes. Chitosomes are intracellular membrane vesicles that host the chitin synthase or its zymogenic precursor form and may contain preformed chitin in their lumen during vesicle transport prior to their fusion with the cytoplasmic membrane [24,25].

Despite its strong ecological impact, the structure of the much more complex insect chitin synthase [26], which is closely related to the C-terminus of the mollusc enzyme [18], has attracted significant research interest in recent years [27]. Mutagenesis experiments and RNAi approaches demonstrated that chitin does not only fulfil a structural role in the arthropod exoskeleton. In fact, it guides the development of invertebrates such as *Drosophila*, *Tribolium*, and *Caenorhabditis *[28-30].

There are several options in order to interfere chemically with the biosynthesis of chitin [31,32]. One prominent example are small-molecule inhibitor drugs such as nucleosid-peptides that structurally imitate the UDP-activated chitin precursor substrate, UDP-N-acetylglucosamine (UDP-GlcNAc) and thus inhibit the chitin synthases of fungi and insects in a competitive manner [33-35]. Polyoxins, first time described in 1965, and Nikkomycins, first described in 1976, belong to this class of inhibitors that are produced by certain strains of *Streptomyces*, such as *S. tendae *and *S. cacaoi *[36,37]. Remarkably, these nucleosid antibiotics did not interfere with protein synthesis (reviewed by [31]). Nevertheless, a broad profile of inhibitory potency, ranging from K_i _= 0.02 μM to K_i _= 3.5 μM, was reported for chitin synthases across different eukaryotic phyla *in vitro *[35]. The commercially available chitin synthase inhibitor NikkomycinZ is well suited for non-invasive cell biological and biochemical studies *in vivo*: It is cell permeable due to active transport mechanisms that are employed by eukaryotic cells for the uptake of dipeptides. The fact that this substance is built from unusual amino acids counts for its resistance against intracellular proteolytic degradation [38-40].

In the early embryonic stages of mollusc development, differentiated ectodermal cells form the shell field, a tissue that excretes the larval shell, as reviewed by Kniprath [41]. In *Mytilus galloprovincialis *the shell field invaginates and forms a 'glandular' canal [42]. The internal lumen of this "shell gland" is closed some hours after fertilization and the outmost cells start to secrete an organic shell cover, the periostracum, underneath a glycocalyx that originates from neighbouring cell microvilli. The "shell gland" everts while forming the larval mantle epithelium and the first mineralized shell [43,44], see [45] for review). The shell of this trochophora larval stage, the prodissoconch I, is enlarged until the entire embryo is covered, and the organism metamorphoses into the motile veliger larva. In bivalves, the subsequently formed shell grows as well in thickness as concentrically at the hinge and at the shell edges, thus forming the prodissoconch II (for explanation, see also Fig. 1 of Ref. [14]). Metamorphosis into the adult stage is indicated by the development of the foot tissue (pediveliger stage), and a sharp transition in microtexture between the remaining larval shell and the newly formed adult dissoconch [46,47]. During the prodissoconch II stage, the shell hinge is elongated in a straight manner. The interlocking hinge teeth are enlarged, and new teeth are formed in a row from the centre towards the edge. Remarkably, the functionality of the hinge apparatus is maintained throughout this complex developmental process [47-49].

**Figure 1:**
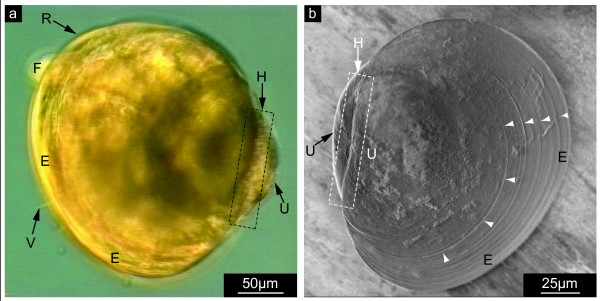
**Shell development of *Mytilus galloprovincialis *larvae under the conditions of the *in vivo *experiments**. The embryos were reared in cultures that were optimized with respect to larval viability, motility, growth, and shell development. **a**. Representative example for larvae grown in 10 l-culture until the stage of the pediveliger/juvenile adult. Metamorphosis occurred within the expected time frame of 31 days as indicated by the well-developed foot (F) and the vanishing velum (V). The edge (E) and its rim (R) are smooth, the hinge or provinculum (H, framed by dotted lines) is straight-lined, and a dorsal protuberance, the so-called umbo (U) is observed as in previous developmental stages (veliger larva). **b**. Scanning electron microscopy image of a shell extracted from a well developed larva at the age of 19 days, previously grown in 40 ml-culture for 11 days. Note the smooth surface of the shell and the regular growth lines (arrowheads).

In a previous study, we investigated the chitinous matrix of larval shells of the marine bivalve mollusc *Mytilus galloprovincialis *[7]. The way how the chitin is distributed throughout the organic framework of the larval shell, how its distribution and structure change with the time-course of larval development, and the reflection of typical functional elements of larval shells such as the hinge teeth, the bivalve symmetry, and the radial and tangential shell growth front within the observed chitin pattern suggest an integrative functional role for chitin synthesis in biomineral composite formation, from the subcellular to the organism scale. These observations motivated us to study the biochemical process of chitin synthesis in relation to the formation of the larval shell. Here, we present the first polarized light microscopic and scanning electron microscopic data from shells of the marine bivalve mollusc *Mytilus galloprovincialis *that were cultivated in the presence of the chitin synthase inhibitor NikkomycinZ.

By using NikkomycinZ *in vivo *as a small-molecule inhibitor drug we demonstrated that even a partial inhibition of chitin synthesis interferes dramatically with the biosynthesis of larval mollusc shells at various hierarchical levels. The presented data suggest that chitin formation may provide one of the regulatory targets of "biologically controlled" [50] mineralization, thus forming a multi-link between the constantly developing mollusc shell and the mantle epithelial cells. This might lead to a novel understanding of the role of the epithelial cells that, at one and the same time, guide larval development, secrete the shell precursor components, and monitor the dynamics of the various shell formation interfaces.

## Results

### Experimental strategy

We chose an *in vivo *approach for investigating the impact of chitin synthesis in mollusc larval shell formation. Therefore, we reared mollusc larvae in the presence of NikkomycinZ, a small-molecule drug that is well-known to inhibit enzymatic chitin synthesis in a competitive manner *in vivo *and *in vitro*. We used polarized light video microscopy for the *in vivo *investigations and scanning electron microscopy imaging of extracted shell material prepared from NikkomycinZ treated *Mytilus galloprovincialis *larvae of various age.

### Shell development under the conditions of the test (control experiments)

The metamorphosis of the mollusc larvae into the pediveliger and subsequently into the adult stage within the expected time frames (Fig. 1a, Additional Files 1, 2, 3, 4, 5, 6, 7, 8, 9, 10) was one of the indications that our established culture conditions meet suitable quality standards for *in vivo *experiments. For control purposes, larvae of *Mytilus galloprovincialis *(Mollusca: Bivalvia) were reared under conditions matching the ones of the test series as closely as possible with respect to culture size, water reservoir, animal density, feeding, light, aeration, water exchange, and temperature, however in the absence of the competitive inhibitor of chitin synthase, NikkomycinZ. The viability as well as the behaviour of the larvae and the development of the larval shell during the treatment was monitored (see Additional Files 5, 6, 7, 8, 9, 10). We found that larvae can be reared in small-scale cultures (1 ml) without any significant loss of viability as compared to 10 l-scale cultures, as long as appropriate concentrations of algae are supplied, and if the culture medium is frequently replaced in order to keep the salt concentration constant and to remove contaminants, excess algae, and larval debris. A scanning electron microscopy image of a well developed, 19-day shell reared under the conditions of the test without NikkomycinZ is shown in Fig. 1b.

### Statistical effects of NikkomycinZ treatment observed in larvae cultures

The effects of NikkomycinZ on the cultivation of larvae were evaluated by estimating the percentage of affected individuals per culture well. It was not possible to determine accurate numbers due to the high motility of the individuals. Based on values obtained from different experimenters, we estimated a deviation of ~20%. This deviation includes also effects of variable numbers of individuals per culture well. We defined test conditions to be appropriate when larvae in the control culture without NikkomycinZ represented viability and motility comparable to a regular 10 l scale culture, which allowed us to grow larvae until metamorphosis into the adult stage occurred. When NikkomycinZ was applied in concentrations of > 25 μM, most of the individuals did not survive for the duration of the test. When NikkomycinZ was applied in concentrations of less than 5 μM, no significant effects on viability or increased morphological changes were observed. We found NikkomycinZ suitable to be applied in concentrations of 5 μM and 10 μM in order to compromise between the detrimental effects on viability and inducing significant effects on shell formation.

**Figure 2:**
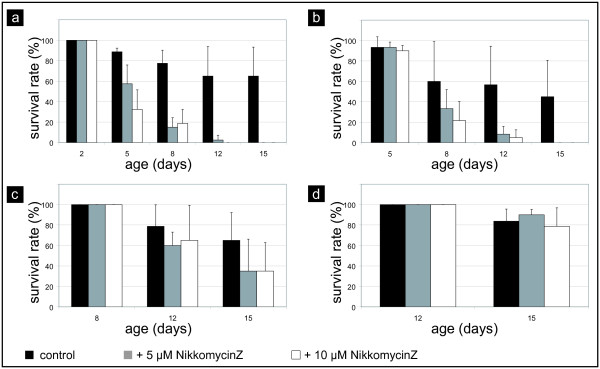
**Viability of *Mytilus galloprovincialis *larvae populations in the presence of NikkomycinZ**. The effect of NikkomycinZ on the viability of larvae was semi-quantitatively estimated based on up to four independent test series that were microscopically evaluated at 80× magnification. The mobility of the larvae prevented an exact quantification of individuals. Based on the comparison of values estimated by different experimenters, the uncertainty was defined as 20% deviation. NikkomycinZ was supplied in concentrations of 0 μM (black bars, control), 5 μM (grey bars), and 10 μM (white bars) at the earliest developmental stages as indicated on the x-axis (larval age in days after fertilization) of each graph. Each test series ended on the 15th day after fertilization. **a-d**. Semi-quantitative estimation of survival rates as observed in larvae test cultures. **a**. Less than 20% of the 2 day old larvae survived a treatment with > 5 μM NikkomycinZ for more than 6 days. **b**. Larvae cultures treated with NikkomycinZ from day five on show a similar effect after three days: The survival rate is about 20% on the 8^th ^day after fertilization in cultures with > 5 μM NikkomycinZ. Exceptionally, this graph represents only three out of four test series as the respective control culture in the 4^th ^series was of low quality. Note that values of more than 100% (deviation bars) are due only to the mean value calculation (see also additional data file 11). **c**. Compared to the untreated larvae populations, about 50% more of the 8 day old individuals died during the following 7 days of NikkomycinZ treatment. Larvae were not as much affected at this age as in younger developmental stages. **d**. The survival rate of 12 day old larvae was not significantly influenced by the addition of NikkomycinZ for three days.

**Figure 3:**
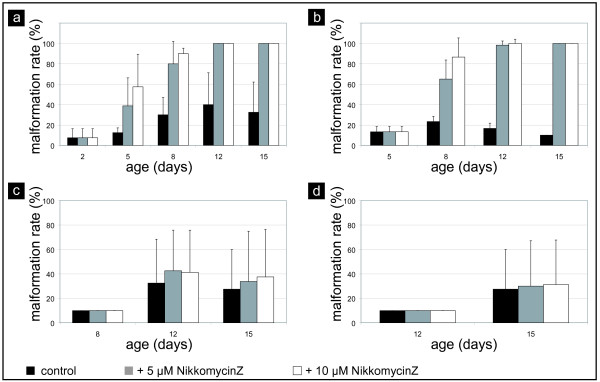
**Malformation rate of *Mytilus galloprovincialis *larvae populations in the presence of NikkomycinZ**. The effect of NikkomycinZ on the development of larvae was semi-quantitatively estimated based on up to four independent test series that were microscopically evaluated at 80× magnification. The mobility of the larvae prevented an exact quantification of malformed individuals (see also supplementary material for video microscopy data). Based on the comparison of values estimated by different experimenters, the uncertainty was defined as 20% deviation. NikkomycinZ was supplied in concentrations of 0 μM (black bars, control), 5 μM (grey bars), and 10 μM (white bars) at the earliest developmental stages as indicated on the x-axis (larval age in days after fertilization) of each graph. Each test series ended on the 15th day after fertilization. **a-d**. Semi-quantitative estimation of morphological abnormalities observed in living individuals of the respective larvae test cultures. The abnormal phenotype rate was defined as 100% once the whole population died. Note that only the most obvious malformations (asymmetric valves, broken shells, "half-naked" individuals) were detected at the scale of binocular microscopy at 80× magnification, whereas for example undulations or tiny fractures of the larval shells were hardly visible. **a**. A steady increase in the abnormal phenotype rate was observed when larvae were grown in the presence of NikkomycinZ from the 2^nd ^day on. About three times more individuals than in the control culture were malformed on the 8^th ^day. **b**. When NikkomycinZ treatment started on the 5^th ^day, the fraction of the abnormal larvae also increased with time. In comparison to the control population, about 50% more individuals were affected after three days. **c, d**. No obvious increase in the malformation rate was observed at 80× magnification for the duration of the test in cultures treated with NikkomycinZ from the 8^th ^day (c) or the 12^th ^day (d) on.

Four independent test series were included in the evaluation. NikkomycinZ was applied in concentrations of 0 μM (control), 5 μM, and 10 μM to 2-day (2d), 5-day, 8-day, and 12-day larvae. The cultures were grown to a final larval age of 15 days (15d). The estimated values for viability and morphological abnormalities of living individuals in each culture are summarized in Fig. 2 and Fig. 3 (see also Additional File 11). Morphological abnormality (Fig. 3) was defined as 100% for a particular culture when no individual survived the treatment. The average percentage of abnormally developed individuals or an increase in mortality in cultures without NikkomycinZ during the observed time span was slightly higher than compared to 10 l cultures due to the higher population density.

As demonstrated in Fig. 2, the early larval stages are more affected by NikkomycinZ treatment than the older larvae. If NikkomycinZ is added to a 2-day culture, the survival rate is below 20% on the 8^th ^day (Fig. 2a). A comparable decrease in the survival rate is obtained when 5-day larvae are treated with NikkomycinZ for seven days (Fig. 2b). 8-day larvae were not affected comparably as much on the average (Fig. 2c). About 50% more individuals than in the control culture died within seven days in the presence of NikkomycinZ. As shown in Fig. 2d, no significant decrease in survival rate was observed during the first three days of NikkomycinZ incubation in 12d old cultures, whereas all younger larval stages did show an effect after three days.

The following criteria were defined in order to classify shell phenotypes that were observed in NikkomycinZ treated cultures: undulated shell edge, bilaterally asymmetrical valves, lack of umbo stage after 12 days, extraordinary small shell relative to the size of the organism, no straight hinge line, increased transparency of the shell, fractured shell. A larval shell was considered affected by NikkomycinZ treatment if one or more of the described phenotypes were applicable. Only motile individuals were taken into account. Fig. 3 shows the percentages of individuals with abnormally developed shells grown in the presence of NikkomycinZ with respect to the criteria described above. With progressive incubation time, the NikkomycinZ treatment from day 2 on induced a steady increase of abnormally developed individuals in the range of three times higher than observed in the control culture (Fig. 3a). Similar results were obtained from cultures incubated from day 5 on. Three days of a treatment with 10 μM NikkomycinZ slightly increased the number of observed shell defects in the population (Fig. 3b). No significant effects of the NikkomycinZ treatment with respect to shell development were observed at the binocular microscope level at 80 × magnification for the older stages of 8–15 days and 12–15 days (Fig. 3c,d).

### Morphological effects of NikkomycinZ treatment on the organism scale (> 100 μm)

**Figure 4:**
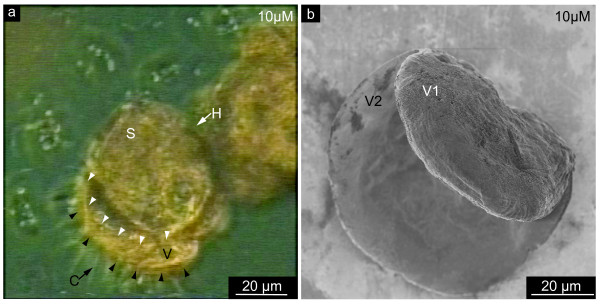
**Phenotype of larval shells synthesized in the presence of NikkomycinZ**. **a**. Video microscopy analysis of a 5 day old larva that has been treated for three days with 10 μM NikkomycinZ. This lively animal synthesized its shell (S) much too small compared to the whole organism. Thus, the shell is not suitable to protect the body tissue. The velum (V, distal border marked by black arrowheads) with its characteristic cilia (C) can not be fully retracted into the shell. The rim of the shell is indicated by white arrowheads. Note that also the hinge (H) of this shell is malformed. **b**. Scanning electron microscopy image of a 5 day old larval shell that consists of asymmetric valves due to 3 days of growth in the presence of 10 μM NikkomycinZ. Note that one valve (V1) is much smaller than the second valve (V2). These data suggest that the inhibition of chitin synthase influences the synthesis and biomineralization of larval shells on length scales of > 100 μm.

It was observed in *Mytilus galloprovincialis *populations grown in the presence of NikkomycinZ that the growth rate of the larval shell is reduced relative to the growth rate of the body tissue. Despite the fact that shells were not adequate in size and thus not suitable to envelope the organism, the animals were still alive and motile as observed by video light microscopy (see Additional Files 11, 12, 13, 14, 15). Fig. 4a shows one representative example (compare also between control and NikkomycinZ treated populations in the video data supplied as Additional Files 5, 6, 7, 8, and 12, 13, 14, 15, respectively). The second main feature observed in NikkomycinZ treated individuals was the asymmetry of the two shell valves. Scanning electron microscopy of shells extracted from NikkomycinZ treated individuals confirmed this impression gained from video microscopy in more detail: the two valves differ significantly in size, thus forming an asymmetric shell where the rims do not fit each other (Fig. 4b). Summarizing the effects on the 100 μm – 1000 μm scale, the asymmetry of the valves, a reduced size of one or both valves relative to the organism's body size, and also a slightly reduced size of the whole individual were among the phenomena observed comparably more often in NikkomycinZ treated cultures.

### Structural effects of NikkomycinZ treatment on the tissue scale (10 μm – 100 μm)

**Figure 5:**
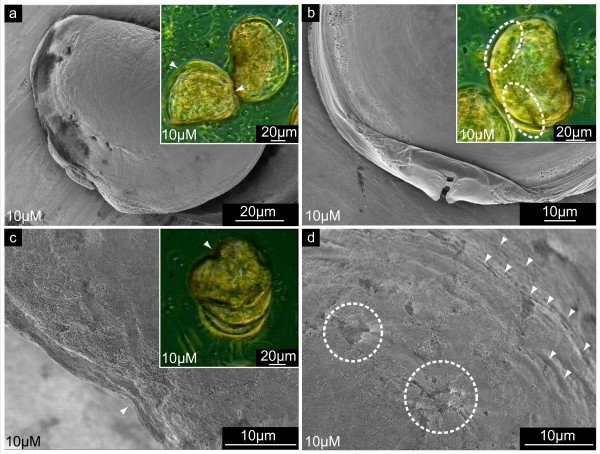
**Phenotype of larval shell parts synthesized in the presence of NikkomycinZ**. **a-d**. Scanning electron microscopy (SEM) images of shells synthesized by larvae exposed to NikkomycinZ at concentrations of 10 μM. All presented features were not observed in any of the shells extracted from the respective control cultures (0 μM NikkomycinZ). Insets show the respective features as observed frequently by video microscopic (VM) *in vivo *analyses of 10 μM NikkomycinZ treated cultures. **a**. Typically, 5 day old shell valves were irregularly wavy edged when larvae were grown in the presence of NikkomycinZ from the 2^nd ^day on. The inset shows the same feature at the age of 8 days in certain shell regions (arrowheads). **b**. If NikkomycinZ was supplied to cultures from the 8^th ^to the 19^th ^day, the larval shell rims and thus the growth fronts are heavily deformed and appear either stretched or squeezed depending on the shell region. The inset shows that features such as undulated shell rims (dotted lines) were observed also in 8 day old larvae that had been treated for three days with NikkomycinZ. **c**. Shells of 8 day old larvae (SEM image) and 12 day old larvae (VM image) that were exposed to NikkomycinZ for three and four days, respectively, appear curved (arrowheads) along the hinge line of the provinculum. **d**. The outer surface of a shell extracted from an 8 day old larva after 3 days of NikkomycinZ treatment revealed local indentations (dotted lines). Note that also the growth lines appear curvaceous (arrowheads). These data suggest that the inhibition of chitin synthase influences the synthesis and biomineralization of larval shells on length scales of 10 μm – 100 μm.

Larval shell valves grown in the presence of NikkomycinZ exhibited drastic abnormalities. As demonstrated in Fig. 5a, the rims (edges) of most NikkomycinZ treated *Mytilus galloprovincialis *shells exhibited undulations. Such undulations were not observed in the control populations cultured in the absence of NikkomycinZ. Especially at the growth front, the rims of the valves appeared deformed or squeezed. In particular cases, the growth lines were curving or appeared rolled-up (Fig. 5b). The hinge line of NikkomycinZ treated animals was not straight but curved or undulated along the provinculum connecting the two valves (Fig. 5c). The development of the umbo stage was usually inhibited or delayed in NikkomycinZ treated larvae; in other words, the shell valves appeared comparably "flat" rather than cone-shaped. The outer surfaces of NikkomycinZ treated larvae showed unusual undulations on the length scale of square microns (Fig. 5d, circles). Growth lines were missing completely or were irregular in shape (Fig. 5d, arrowheads; compare control specimen in Fig. 1b).

### Ultrastructural effects of NikkomycinZ treatment on the subcellular to cellular scale (<1 μm – 10 μm)

#### Inner surface layer – lateral growth and thickening of the larval shell

**Figure 6:**
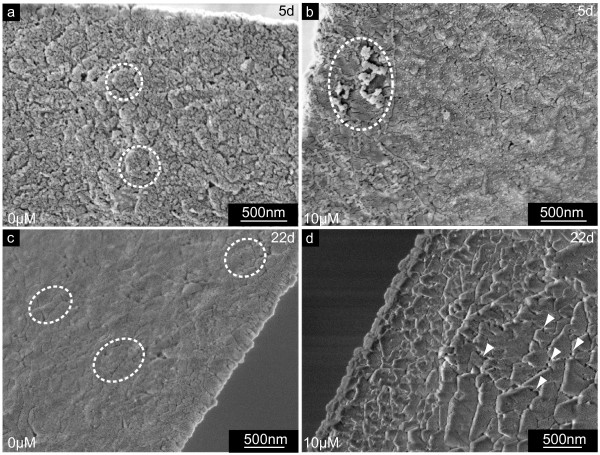
**Larval shell inner surfaces synthesized at the distal growth front in the presence of NikkomycinZ**. Scanning electron microscopy (SEM) images of shell specimens synthesized by mollusc larvae in the presence (10 μM) or absence (0 μM) of NikkomycinZ. The distal growth at the shell edges of 5 day old (a,b) and 22 day old (c,d) specimen is shown. Note the difference in smoothness and compactness between the 3 days (a,b) and 10 days (c,d) NikkomycinZ treated (b,d) and control (a,c) cultures. **a**. A well-developed shell is covered by flakes (dotted lines) that consist of a granular material. This shell layer is veiled by another fraction of fine dispersed material. **b**. NikkomycinZ treated larvae produce hackly shaped shell edges with irregular deposition of material on the inner surface (dotted line). **c,d**. At later developmental stages (22 days), well-developed shells (c) are more compact but still resemble the shell surface structure of the 5 day old control larva (a) in terms of the flakes (dotted lines) and the fine dispersed material fraction, whereas NikkomycinZ treatment causes the formation of irregular and hackly shaped rims at the shell's edge (d). The lack of fine dispersed material reveals interspaces with thin connections between the flakes (white arrowheads). Note that the size of the flakes decreases towards the edge. These data suggest that the inhibition of chitin synthase influences the lateral growth and may influence phase transitions in the biomineralization of larval shells on length scales of < 1 μm – 10 μm.

The shell edge of *Mytilus galloprovincialis *larvae usually appears smooth and compact. As demonstrated in Fig. 6a, the inner shell surface appears to consist of submicron flakes, covered by a different kind of fine dispersed granular material on the surface. The inner surface of shells extracted from larvae grown in the presence of NikkomycinZ appeared irregular and hackly shaped (Fig. 6b). Additional irregular agglomerates (Fig. 6b, dotted lines) were associated with the inner shell surface. These phenomena were predominantly observed at the newly formed shell edge. In principle, the same features as described for 5 day old larval shells (Fig. 6a,b) are present, and even more pronounced in 22 day old shells (Fig. 6c,d). The edge of shells extracted from an untreated control population (Fig. 6c) is smoothly fringed, and the material appears compact from the shell's centre to the rim. Again, the material consists of flakes that fit to each other like pieces of a puzzle. The surface is still covered by a fine dispersed material. NikkomycinZ treated 22 day old larvae formed valves with irregular rough margins (Fig. 6d). The inner surface was covered with square-edged flakes. It was observed that the size of these flakes decreases towards the shell margin, which indicates either the interference of NikkomycinZ with at least two different growth mechanisms (lateral growth, thickening), or a variation in the effective concentration of NikkomycinZ (see discussion for details).

These shells (Fig. 6d) also lack the granular layer, which usually covers the flake-like material on the inner shell surface (also compare Fig. 6c). The uncovered flakes (Fig. 6d) have evident interspaces between them, which also decrease towards the shell edge.

**Figure 7:**
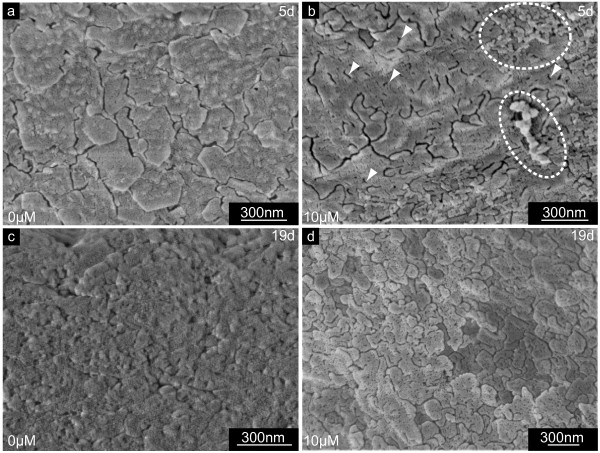
**Shell thickening in the presence of NikkomycinZ**. Scanning electron microscopy (SEM) images of shell specimens synthesized by mollusc larvae in the presence (10 μM) or absence (0 μM) of NikkomycinZ. Regions of shell thickening of 5 day old (a,b) and 19 day old (c,d) specimen are shown. Note the difference in smoothness and compactness between the 3 days (b) and 11 days (d) NikkomycinZ treated and the respective control cultures (a,c). **a**. The central inner surface of a 5 day old control shell reveals flakes with a homogeneous, fine-disperse granular surface structure. **b**. Irregular shell material agglomerated on top of the flakes (dotted lines) in NikkomycinZ treated organisms. Individual flakes bared an internal slit-like nano-scale structure (arrowheads). **c**. The inner surface flakes are more confluent in later developmental stages. The surface appears more compact, smooth, and homogeneous. **d**. After 11 days of growth in the presence of NikkomycinZ, the inner surface structure of larval shells in principle resembles the flake-like structure as observed in shells of NikkomycinZ treated younger developmental stages (b). These data suggest that the inhibition of chitin synthase influences the growth in thickness and biomineralization of larval shells on length scales of < 1 μm – 10 μm.

Fig. 7a–d show the inner surface of central parts of shells of 5 day old (Fig. 7a,b) and 19 day old (Fig. 7c,d) larvae, respectively. In these areas, the NikkomycinZ treatment (Fig. 7b,d) affected predominantly the surface of the material. The overall structure of the inner shell's surface did not differ significantly from the control. However, the fine structure of the individual flake's surface did: As demonstrated for a 5 day old larval shell in Fig. 7a, the surface texture of the flakes formed without NikkomycinZ appears fine-dispersed granular and compact. When the larvae were grown in the presence of NikkomycinZ from day 2 on, the inner surface of the 5 day old larval shells showed irregular agglomerates (Fig. 7b dotted lines) that were associated with the shell's surface. The shell flakes (several hundreds of nm in diameter) were interspersed with numerous slits of a few nm in diameter (Fig. 7b, arrowheads). In later developmental stages the inner surface of larval shells is usually smooth, homogeneous, compact, and the shell flakes are more confluent than in the younger developmental stage. This is demonstrated in Fig. 7c for a 19 day old individual. Once larvae were grown in the presence of NikkomycinZ from day 8 on until the same age (Fig. 7d), their shells exhibited a porous and flake-like structure on their inner surface, which is structurally comparable to the one described for the 5 day old larvae treated with NikkomycinZ from day 2 on (Fig. 7b). It can be concluded from these results that a partial inhibition of chitin synthesis by NikkomycinZ influences not only the lateral growth and the thickening of the larval shell, but also the fine-tuning of interfaces of macromolecular components that aggregate with the mineral phase into the final shell.

#### Outer surface layer – interference of NikkomycinZ with periostracum formation

**Figure 8:**
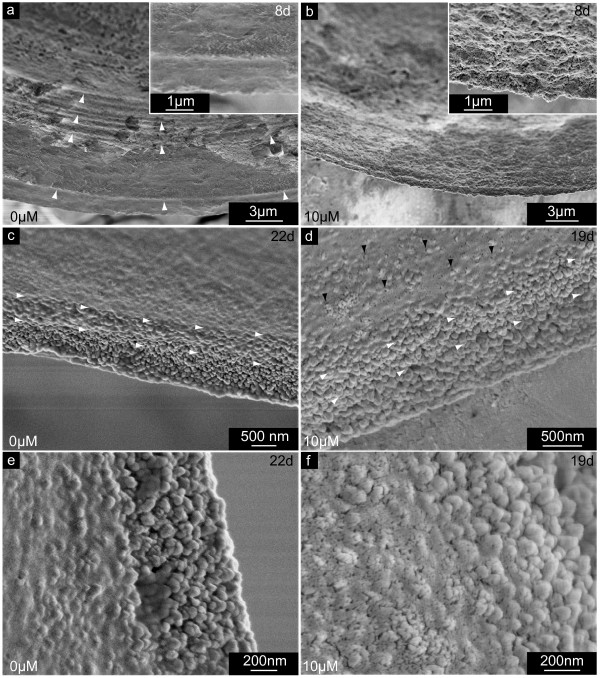
**Outer surfaces of larval shell edges synthesized in the presence of NikkomycinZ**. Scanning electron microscopy (SEM) images of shell specimens synthesized by mollusc larvae in the presence (10 μM) or absence (0 μM) of NikkomycinZ. Shell edges of 8 day old (a,b) and 19 day old (d,f), and 22 day old (c,e) specimen are shown. Note the difference in smoothness and compactness between the 3 days (b) and 11 days (d,f) NikkomycinZ treated cultures, and the respective control cultures (a,c,e). **a**. In control cultures, the prodissoconch II shows regular growth lines (arrowheads) and a smooth shell rim. **b**. Three days of NikkomycinZ treatment produced irregular shell edges in eight day old larvae. Defined growth lines are not present. The inset shows a close-up view of the irregular, porous surface texture. **c**. The shell edge of a 22 day old control larva consists of globular elements. The globules are embedded within a homogeneous layer in the previously formed shell parts (arrowheads). **d**. The outer surface has a porous appearance (black arrowheads) in 19 day old shells formed for 11 days in the presence of NikkomycinZ. The homogenous shell surface layer is not as distinct as observed in control cultures (compare white arrowheads in (c) and (d)). **e**. A higher magnification image of (c) shows in detail the transition region between the shell rim and the outer surface region covered by the homogeneous layer. **f**. Image of the specimen described in (d) at the same scale and at a comparable shell position as the control (e). Note the differences in the texture and porosity of the surface layer. These data suggest that the inhibition of chitin synthase influences the outer shell cover and biomineralization of larval shells on length scales of < 1 μm – 10 μm.

The outer surface of *Mytilus galloprovincialis *larval shells appears smooth in the prodissoconch I stage, whereas the prodissoconch II exhibits regular growth lines, and a well-defined, smooth edge (Fig. 8a). If larvae are exposed to NikkomycinZ, the appearance of the growth lines of the outer surface of prodissoconch II changes. This is shown in Fig. 8b. At higher magnification, the material deposited in the presence of NikkomycinZ at the shell's edge by an 8 day old larva reveals irregular growth lines and a porous fine structure (Fig. 8b, inset). In older stages of shell development, the influence of chitin synthase inhibition by NikkomycinZ is predominantly apparent at the shell's edge. The edge of untreated shells consists of globular elements that are regularly deposited on the outer shell surface on top of an inner layer. The particles closest to the shell edge appear embedded into the inner, homogeneous layer. As demonstrated in Fig. 8c, the globular particles are covered by a fine-dispersed homogeneous layer in the previously formed shell parts (Fig. 8c, arrowheads). Fig. 8d demonstrates that this fine-dispersed layer is not completely homogeneous, but porous in the shells of NikkomycinZ treated larvae cultures. The respective close-up views show more clearly the differences in the fine structure of this layer formed in the absence (Fig. 8e) and presence (Fig. 8f) of NikkomycinZ. It has to be taken into account that the appearance of these layers is influenced to a certain extent by partial etching, due to an exposure to deionised water during the preparation for SEM imaging. There is also no protection against etching by the periostracum, which has been removed during the previous sodium hypochlorite extraction of larval shells. These observations suggest that even the formation of the layer next to the periostracum is in some way influenced by the NikkomycinZ inhibited chitin synthesis. When NikkomycinZ was applied to larvae cultures in the very early veliger stage, the structure of the outer surfaces of both, prodissoconch I and II were affected.

#### Hinge formation and hinge ultrastructure

**Figure 9:**
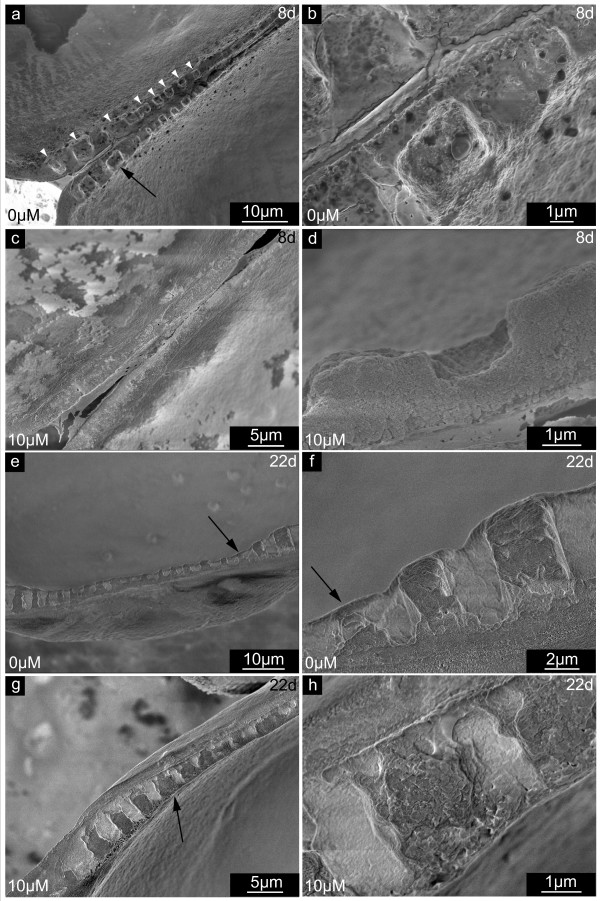
**Phenotype of larval shell hinges synthesized in the presence of NikkomycinZ on length scales of < 1 μm – 100 μm**. Scanning electron microscopy (SEM) images of shell specimens synthesized by mollusc larvae in the presence (10 μM) or absence (0 μM) of NikkomycinZ. Hinge regions of 8 day old (**a-d**) and 22 day old (**e-h**) shell specimens are shown. **a**. Hinge teeth (arrowheads) are periodically distributed. The size of individual hinge teeth decreases towards the centre of the hinge. **b**. A close-up view of the hinge tooth marked by an arrow in (a) reveals the compact, cubic shape and the smooth, granular surface structure. **c**. Provinculum of a larva cultured for three days in the presence of NikkomycinZ that lacks well developed hinge teeth. This view represents the inner surface of the valves, where usually hinge teeth or their precursors are observed. **d**. Fragments of hinge teeth of an eight day old larva cultivated in the presence of NikkomycinZ. The teeth are not cubic and there is no granular cover layer. **e**. In later developmental periods, the hinge teeth are regularly distributed along the total length of the hinge. Note that even the youngest formed tooth precursors located in the centre of the hinge built a protuberance. **f**. Close-up view of the transition region between the bigger and a more recently formed smaller tooth as indicated by an arrow in (e), revealing the fine-grained cover layer and the smoothly curved edges. **g**. Hinge of a larva that have been reared in the presence of NikkomycinZ since the 12^th ^day after fertilization. The smallest teeth were flat (or missing) and the development of the most recently formed teeth appeared delayed in comparison to untreated larvae cultures. **h**. Close-up view of the transition region as indicated by an arrow in (g) between the earlier and recently formed hinge teeth. The teeth appear compressed and the surface is covered with flat, sharp-edged platelets.

A straight hinge line is characteristic for healthy bivalve larvae. We observed that a big proportion of individuals that grew in the presence of NikkomycinZ revealed a curved hinge (Fig. 5c). The formation of the hinge teeth was affected by NikkomycinZ as demonstrated in Fig. 9. The hinge of a larval shell grown in the absence of NikkomycinZ contains up to 24 teeth periodically arranged in two rows, one per valve, on the 8^th ^day after fertilization (Fig. 9a). Each hinge tooth has a compact cubic shape with a smooth, granular surface structure (Fig. 9b). The size of the hinge teeth varies from the centre of the hinge line towards the edge. When NikkomycinZ was added to the culture medium on day 5, the hinge of 8 day old larvae did not develop well-shaped hinge teeth. An extreme example of one specimen without any visible hinge teeth is shown in Fig. 9c. The hinge teeth of another 8 day old larvae grown in the presence of NikkomycinZ are shown in detail in Fig. 9d. They appear like the fragments of hinge teeth, probably due to either an increased dissolution of shell material or limited deposition of chitin, and thus limited deposition of other shell material.

The influence of NikkomycinZ on the development of hinge teeth is strongly dependent on the time frame of NikkomycinZ application. In older developmental stages, hinge tooth formation is not completely inhibited by NikkomycinZ. However, we observed structural alterations that might predominantly affect the functionality of the youngest formed hinge teeth. For comparison, the hinge of a larval shell from the control culture without NikkomycinZ is shown in Fig. 9e. Even the youngest teeth in the centre of the hinge represented at least small protuberances. The transition region between the smaller (Fig. 9e, arrow) and the bigger hinge teeth is shown in detail in Fig. 9f. All teeth exhibited smoothly curved edges. Each tooth spans the whole cross-section (thickness) of the shell's hinge throughout the complete line. The picture changes, once NikkomycinZ was present in the culture medium during shell growth (Fig. 9g,h). It is obvious that especially the smaller hinge teeth (Fig. 9g) were not well formed. As observed more clearly at higher magnification (Fig. 9h, arrow indicates the zoom-in position in Fig. 9g), such hinge teeth did not span the whole thickness of the shell. The edges of the hinge teeth did not confine cubic elements. The same structural features applied to the bigger hinge teeth. The close-up view of the small hinge teeth (Fig. 9h) revealed that the building blocks of the hinge region in larvae grown in the presence of NikkomycinZ consisted of small, flat, and sharp-edged prismatic building blocks with a planar upper surface. Such sharp-edged building blocks were never observed in hinges of control larvae, which were smoothly curved-edged, and which were equipped with a fine-dispersed globular surface cover (Fig. 9b,f). These results indicate a direct interference of NikkomycinZ with the formation and remodelling of hinge teeth in the bivalve larvae of *Mytilus galloprovincialis *throughout development.

### Effects of NikkomycinZ treatment on the crystallization and molecular self-assembly scale (Å – 100 nm)

**Figure 10:**
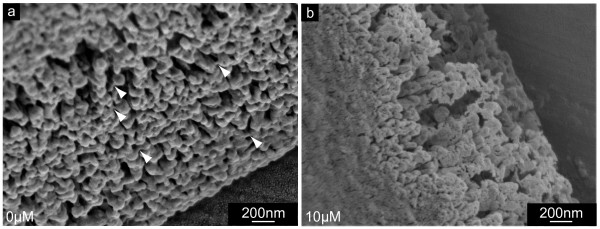
**Solubility of larval shells synthesized in the presence of NikkomycinZ**. Scanning electron microscopy images of shell specimens that appeared affected by an exposure to distilled water. This treatment is known to partly dissolve the amorphous calcium carbonate fraction of the mineral phase. **a**. A 19 day old larval shell that was synthesized in the absence of NikkomycinZ is shown for comparison. The remnants of the outer shell edge consist of a pattern of columnar depositions with thin filigree-like connections between each other (arrowheads). **b**. NikkomycinZ treatment of mollusc larvae for 11 days prior to shell extraction at the 19th day reveals that the shell material is more sensitive to the etching by deionised water. The shell edge appears much more porous and the columnar depositions are irregular.

Several observations indicated that the effects of the chitin synthase inhibitor NikkomycinZ did not only cover the length scales of the organism, organ, tissue, cells, and subcellular compartments, but as well the mineralization process. Several prominent features have been discussed in the previous section, such as the sharp-edged appearance of building blocks of the hinge teeth (Fig. 9h). The presence of flat prisms in NikkomycinZ treated animals (Fig. 6d, Fig. 9h) instead of granular material indicates a strong interaction between the NikkomycinZ sensitive biochemical synthesis pathways such as chitin synthesis and the way of mineral deposition and crystallization. In the following, two additional key observations with respect to shell mineralization are presented: The first one refers to structures that were obtained presumably due to partial dissolution of the mineral phase in distilled water, to which the samples were exposed during the sample preparation for scanning electron microscopy. The dissolution process apparently affected larval shell preparations from NikkomycinZ treated and non-treated cultures, each in a different manner. An example for a non-treated 19 day old shell is given in Fig. 10a. The outer surface of the shell edge consists of columnar depositions that are arranged in a regular manner. The surface of each column is smooth, and each column or stalk is terminated by a globular structure (~50 nm in diameter). The columns or stalks are bridged between each other (Fig. 10a, arrowheads) by elongated structures that appear to exist separately without any stalk. The hollow space between the columnar structures indicates that the original shell texture was altered by extended etching in deionised water (for comparison, see Fig. 8e). Comparably dissolved edges of shells extracted from NikkomycinZ treated cultures appeared differently (Fig. 10b). No regular columns were observed, and neither smooth globular edges, nor elongated bridges were present. The overall appearance of the dissolved shell edge was irregular and porous. The porosity of each particular columnar equivalent in Fig. 10b to the smooth globular edged columns in Fig. 10a appears self-evident. Conceivably, the dissolution process seems to affect the mineral composite structures obtained in the presence and absence of NikkomycinZ each in its own way. The formation process and, conceivably, the materials properties of the two mineral composites are therefore different. As shown in Fig. 9d, even the hinge region is probably more sensitive to dissolution due to NikkomycinZ treatment during its formation.

**Figure 11:**
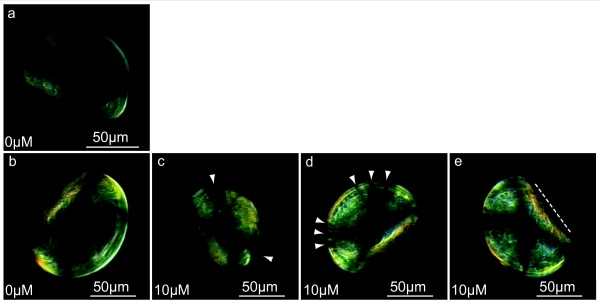
**Crystallization of larval shells synthesized in the presence of NikkomycinZ**. Polarized light video microscopy *in vivo *study of mollusc larvae. Note that the observed colours cannot be assigned purely to the birefringence of the shell as larval shells are usually transparent at the investigated developmental stages. The dynamics of the larval tissue also causes light scattering. Thus, alterations of the birefringence pattern have to be taken into account. **a**. Almost no birefringence was observed in a 2 day old organism, which is consistent with a high fraction of amorphous calcium carbonate in the shell (Prodissoconch I). **b**. A normally developed larva at the age of 5 days is shown. In general, the prodissoconch II contains a higher fraction of crystalline shell material and is therefore more birefringent than a two day old larva. The characteristic dark cross indicates a radial arrangement of the aragonite crystals. **c**. 5 day old larva grown in presence of NikkomycinZ for three days. Here, the shell is too small in comparison to the organism. The characteristic dark cross is visible (arrowheads). The intensity of the birefringence is higher compared to the control organism shown in (b). **d**. 5 day old larva grown in presence of NikkomycinZ for three days. This individual is representative for larval shells that appeared well developed in bright field microscopy. Crossed polarizer images revealed that such larval shells often exhibit a "split-up" dark cross (arrowheads). **e**. NikkomycinZ treated 5 day old larva with a curved hinge (see dotted straight line next to the hinge for comparison). The dark cross appears sharper than that of untreated larva in (b). NikkomycinZ treated larvae appear in general more birefringent. Note also the comparably smaller shell size of the NikkomycinZ treated larvae (c-e).

The second key observation refers to polarized light microscopic analyses of NikkomycinZ treated larvae (Fig. 11). Almost no birefringence is observed in 2 day old larval shells (Fig. 11a) due to the fact that the shell consists mainly of amorphous calcium carbonate. The increased birefringence of a 5 day old larval shell that was grown in the absence of NikkomycinZ is shown in Fig. 11b. Three major shell phenotypes with respect to birefringence were observed in larvae that were grown in the presence of NikkomycinZ (Fig. 11c–e). As demonstrated in the polarized light microscopy image (Fig. 11c), larval shells that were too small compared to the size of the organism ("half-naked" bivalve) exhibited the characteristic dark cross (Fig. 11c, arrowheads), which indicates a more or less radial arrangement of crystals in the larval shell [51]. The overall birefringence, and thus the crystallinity of such shells was comparably high, taking into account that this shell part represents to a large extent the first formed shell (prodissoconch I) which is supposed to consist of a large fraction of stable amorphous calcium carbonate (ACC) (see Fig. 11a for comparison with a 2 day old control larva, and the central shell part (prodissoconch I) of the 5 day old larva in Fig. 11b). With respect to the highly irregular shape of such shells, this result suggests that the inhibition of chitin synthesis by NikkomycinZ does not necessarily interfere with the initial deposition (radial arrangement) of crystals, but rather with the local stabilization or controlled transformation of ACC into aragonite. A second characteristic birefringence phenotype of an otherwise "healthy" looking shell was the split-up appearance of the dark cross (Fig. 11d, arrowheads), which could either be an artefact due to NikkomycinZ induced shell undulations, or indicate that the undulations are caused by NikkomycinZ induced alterations in the crystallization process. Both, the increased birefringence and the splitting of the dark cross were observed as well in shells exhibiting a curved hinge due to NikkomycinZ treatment (Fig. 11e). In general, the shell size of 5 day old NikkomycinZ treated larvae (Fig. 11c–e) was comparable to 2 day old untreated larvae (Fig. 11a) and thus smaller than the regular size of 5 day old untreated larvae (Fig. 11b). Although we currently lack a detailed comparison of cross-sections, our visual microscopic impression was that shells of NikkomycinZ treated larvae appeared more transparent, or thinner than shells of control animals.

### Effects of NikkomycinZ treatment on the physical properties and functionality

**Figure 12:**
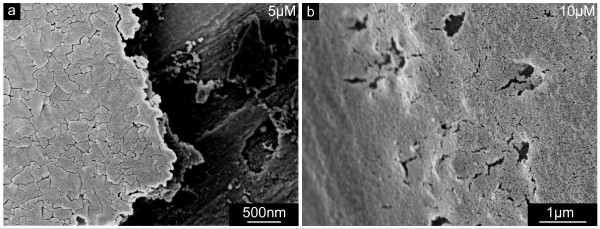
**Mechanical integrity of larval shells synthesized in the presence of NikkomycinZ**. Scanning electron microscopy images of shells extracted from NikkomycinZ treated larvae. **a**. The inner shell surface of a 5 day old larva grown in the presence of 5 μM NikkomycinZ for three days appears brittle along the borders of the flakes. **b**. Holes were observed in the outer shell surfaces of larvae. Here, one 19 day old example is shown. The shell was extracted after the larva was treated with 10 μM NikkomycinZ from the 8^th ^day on.

From all the previously described structural phenomena it can be deduced that NikkomycinZ affects shell formation and thus also the functionality of the larval shell. One of the key properties of mollusc shells is their remarkable fracture resistance compared to the quantity of material used. Even though chitin is not the only component responsible for the integrity of this complex composite ceramic material, it appears reasonable that the synthesis of chitin is coordinated with the secretion of other organic and inorganic shell precursors. Therefore, it is unsurprising that the fragility of larval shells grown in the presence of NikkomycinZ is increased, as shown in Fig. 12. The break lines of such shells follow the borders of the puzzle flakes as demonstrated in Fig. 12a. Furthermore, the outer shell surface appears brittle. This brittleness may also induce the formation of holes that were observed in some shell regions (Fig. 12b).

## Discussion

The primary aim of this study was to analyze the impact of chitin synthesis on mollusc shell formation. If the cellular regulation of chitin synthesis is related to mollusc shell formation, the partial inhibition of enzymatic chitin synthesis by a competitive enzyme inhibitor such as NikkomycinZ is supposed to induce structural alterations in the shell. Since also mollusc larvae of the marine bivalve species *Mytilus galloprovincialis *express the gene for a myosin chitin synthase specifically in the shell forming tissue [18], and since larval shells of this species consist of considerable amounts of chitin [7], these larvae provide an ideal system for studying possible effects of NikkomycinZ on the development of mollusc shells. Especially effects related to the transformation of amorphous calcium carbonate (ACC) into aragonite can be observed more clearly during larval shell development [14], while providing the option to extrapolate the results for a better understanding of the adult shell formation process [3]. The study of larval shell formation processes is of fundamental interest since both, the mineralogy and the structural organization of the larval shell, are evolutionary highly conserved among diverse bivalve taxa. The fact that a "classification of larvae by hinges results in a classification closely parallel to a classification of adults" as recognized by Rees in 1950 [48] indicates that the development and biomineralization of the hinge (provinculum) is of special evolutionary interest. The results presented in this study indicate that NikkomycinZ predominantly interferes with the mineralization process at the hinge region, suggesting that the mollusc myosin chitin synthase may have evolved as an effective regulatory element between cell differentiation and tissue mineralization.

Chitin synthesizing enzymes are complex transmembrane proteins that, unlike other glycosyltransferases [52], have an evolutionary highly conserved amino acid motif in their intracellular active site, which is identical in unicellular fungi as well as in more complex organisms (arthropods, molluscs), and which is well-known to accept UDP-GlcNAc as the only substrate [27]. Conceivably, chitin synthases are highly sensitive for the structural UDP-GlcNAc-analogue NikkomycinZ that inhibits chitin synthesis in a competitive manner and is therefore applied as a pesticide against fungi and arthropods [32]. This drug was also used to test the biological impact of the insect chitin synthase and found to be lethal for first instar larvae at their transition to second instar at concentrations of > 1 μM [26]. It is self-evident that NikkomycinZ can be applied for structural studies on mollusc shell formation *in vivo *in only sublethal concentrations. We determined a suitable working concentration of NikkomycinZ in the range of 5 μM to 10 μM for larvae of *Mytilus galloprovincialis*. It is important for the interpretation of the obtained results to keep in mind that chitin synthesis is not supposed to be completely inhibited in that concentrations range. The exact degree of enzyme inhibition can hardly be estimated *in vivo*, as the quantity and enzymatic activity of mollusc chitin synthases could not be determined, and the exact rate of NikkomycinZ uptake into the larval tissue by mollusc peptide transport systems is unknown. Nevertheless, a phenomenological comparison between larvae cultured in the absence and presence of NikkomycinZ revealed that even a partial inhibition of chitin synthesis at 5 μM – 10 μM inhibitor induced drastic structural alterations in the larval shells. Taking the limited stability of NikkomycinZ in the culture medium (sea water) into account [53], it can be assumed that the effective working concentration of NikkomycinZ continuously decreased from the start value at the time of sea water replacement to the next sea water exchange, allowing the animals to compensate temporarily the detrimental effects on cell metabolism. In fact, the calnexin and calreticulin pathways [54,55] provide eukaryotic cells with effective mechanisms to circumvent detrimental effects that an activated sugar analogue such as NikkomycinZ might have on the posttranslational modification of glycoproteins [56]. Still, it has to be taken into consideration that certain extracellular biomineralization proteins, which are typically glycosylated (see [57] for review), might have been affected by NikkomycinZ as well, and therefore may contribute to certain effects on shell formation observed in this study. Furthermore, an altered level of chitin deposition could well interfere with intracellular signal transduction pathways that regulate the expression of biomineralization genes.

We optimized the NikkomycinZ treatment of the mollusc larvae in order to maintain a survival rate as documented in Fig. 2. Due to the fact that also chitin synthesis was not intended to be completely inhibited, the culture may have been kept under conditions that allow proteins glycosylation to occur above the critical threshold. We observed that even those organisms were extremely vital that were only partly covered with a shell. Therefore, we concluded that chitin synthesis is the much more NikkomycinZ sensitive process. Schlüter dealt with the same problem many years ago [58], inspired by the observations of Holst and colleagues that Nikkomycin was predominantly lethal for arthropod larvae in the course of moulting [59]. In-between the moulting cycles, glycoproteins must be equally important for the survival of the larvae. Obviously, the synthesis of glycoproteins was significantly less affected in these invertebrate animals. Schlüter observed Nikkomycin induced ultrastructural defects in the procuticular region in moulting beetle larvae. Thus, the enzymatic synthesis of chitin appears to be a rather specific target of the Nikkomycins.

One of the general problems of experiments with mollusc larvae cultures is that a 100% survival rate can never be achieved, due to the fact that with proceeding development, a genetic defect of any individual might become lethal or may at least lead to an abnormal phenotype. Therefore, the really significant effect of NikkomycinZ on mollusc larvae populations was that after a certain period of incubation time, none of the animals had a "healthy" phenotype such as observed in populations that have not been in contact with the drug. Malformed animals were not always completely detected as such, mainly due to the low resolution of binocular microscopy, and due to the high motility of the individual larvae and the movements of velar tissue and cilia (see also Additional Files 5, 6, 7, 8 and 12, 13, 14, 15). Therefore the numbers of abnormalities within a NikkomycinZ treated population may in reality be higher than estimated (see Fig. 2 and Fig. 3 for details). Certain malformations were detectable in a definite way only by using scanning electron microscopy, which was not applicable for an *in vivo *screening.

We regarded it important to study morphological effects of the NikkomycinZ treatment by video microscopy. This method allowed us to demonstrate the viability of the organism along with the induced morphological alterations. Our primary aim was to exclude those individuals from the analysis that were affected by NikkomycinZ in any respect other than shell formation. On the other hand, it is conceivable that malformed shells will also interfere with the regular development of a bivalved organism. Therefore, the criteria as described in the results section were defined in order to group the phenotypes observed only in the motile organisms. In later experimental stages, shells of lethargic organisms were also taken into consideration, if a certain morphological effect was exposed there more clearly.

During the time-course of this study, it was recognized that certain categories of malformations were visualized by video microscopy only in a later developmental stage (after a longer treatment with NikkomycinZ), whereas the same features were observed by SEM comparably earlier (after short treatment with NikkomycinZ). This phenomenon becomes especially clear in Figure 5, when comparing the respective age of specimens in SEM and video microscopy images. Undoubtedly, this is one of the limitations of the *in vivo *approach on shell formation. Nevertheless, it can be speculated that the number of individual shells affected by NikkomycinZ was even higher than suggested by the data presented in Figure 3.

**Figure 13:**
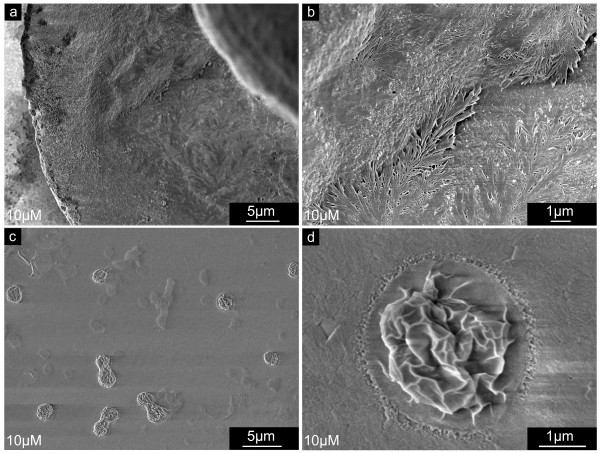
**Rare phenotypes of larval shell inner surfaces synthesized in the presence of NikkomycinZ**. Scanning electron microscopy images of characteristic inner shell surface features that were observed repeatedly, but exclusively each in one particular larva. **a**. The inner shell surface of a 5 day old larva grown in the presence of 10 μM NikkomycinZ from the 2^nd ^day on exhibited "ice-flower" like structures. These characteristic structures were distributed all over the shell. **b**. As demonstrated by the higher magnification view of (a), the borderless transition between the "ice-flowers" and the surrounding flat shell regions exclude the possibility of recrystallization artefacts introduced by sample preparation. **c**. "Rosette-like" structures as shown here were found distributed all over the inner shell surface of a 22 day old larva that had been treated with 10 μM NikkomycinZ for 10 days. **d**. Higher magnification view of (c) reveals that the observed "rosettes" can not be assigned to etching by deionised water due to their direct integration into the larval shell surface. Note that none of the described features or anything comparable was observed in larvae without NikkomycinZ treatment. These rare, individual specific, but characteristic shell features may be indicative for a link between chitin synthase inhibition by NikkomycinZ and additional biomineralization factors. These data suggest that the investigated individuals may have non-lethal mutations in one or more of the additional biomineralization genes.

The interpretation of the observed phenomena is a tightrope walk: At first glance, our results suggest a direct impact of NikkomycinZ on chitin synthesis, despite taking some general effects on protein glycosylation [56] into account. However, as cellular regulation pathways are non-linear in general, which may apply for chitin synthesis in particular, one should keep in mind that effects are manifold and various effects may combine. Based on these considerations, several options remain for interpreting the observed shell morphologies in NikkomycinZ treated *Mytilus galloprovincialis *larval populations: The list of possible effects during chitin synthesis includes various interference patterns such as 1) with chitin fibril formation, 2) with the quantity of chitin in relation to the secreted mineral, 3) with local differences in the quantity of chitin deposition, 4) complex regulatory effects up to interference with genetic feedback-loops and signalling pathways of shell mineralization including shell proteins. This points towards the biggest problem of an *in vivo *approach in understanding the role of chitin synthesis in the regulatory network of mollusc shell formation: The function of many mollusc shell proteins still remains unknown [57], which is partly due to the fact that their activity depends on concerted interactions [60]. Provided that artefacts (e.g. induced by drying of SEM samples) can be excluded, a possible explanation for some very rarely observed shell malformations in NikkomycinZ treated larva as shown in Fig. 13 could be that one or more additional, though unknown key players in biomineralization are mutated. The combined effects may then lead to uncontrolled self-assembly and mineralization and the organism may try to compensate the detrimental effects at different hierarchical control levels (for example, compare Fig. 13a, and 13b).

**Figure 14:**
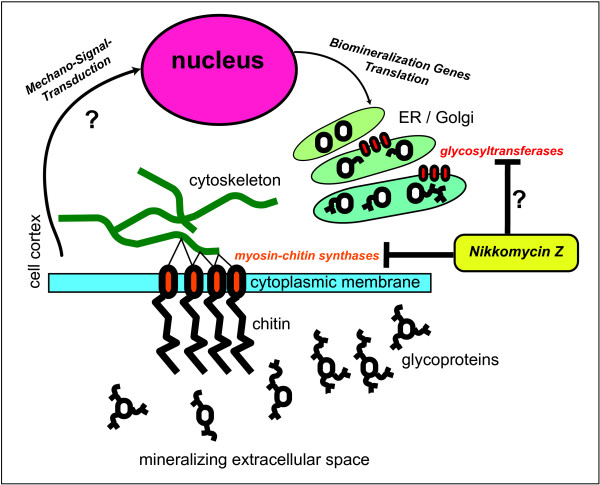
**Putative mechanistic hypotheses for the interference of Nikkomycin Z with mollusc shell formation**. Schematic representation of possible modes of action of NikkomycinZ that are consistent with the observed effects on shell mineralization. In mollusc mantle cells, NikkomycinZ unequivocally inhibits chitin synthesis, whereas the extent of inhibition of glycosyltransferases involved in posttranslational protein modification is currently unknown. The proper glycosylation of secreted proteins is subject to cellular control mechanisms. However, biomineralization is an extremely fine-tuned process that is highly sensitive to the glycosylation pattern of the proteins involved in crystal nucleation and growth. Thus the NikkomycinZ induced shell malformations must not purely be associated with chitin synthesis inhibition. A putative mechanical feedback loop mediated by the myosin coupling of the chitin synthase with cytoskeletal signal transduction could explain the complexity of observed effects induced by NikkomycinZ, such as differences in intensity and quality of effects (survival and malformation rates, mineral texture, shell solubility etc.) observed in larvae of various age.

We summarized the possible consequences of NikkomycinZ in the shell formation process of molluscs in a schematic model (Fig. 14). In mollusc mantle cells, NikkomycinZ unequivocally inhibits chitin synthesis, whereas the extent of inhibition of glycosyltransferases involved in posttranslational protein modification is currently unknown. The proper glycosylation of secreted proteins is subject to cellular control mechanisms. However, biomineralization is an extremely fine-tuned process that is highly sensitive to the glycosylation pattern of the proteins involved in crystal nucleation and growth. Thus the NikkomycinZ induced shell malformations must not purely be associated with chitin synthesis inhibition. A putative mechanical feedback loop mediated by the myosin coupling of the chitin synthase with cytoskeletal signal transduction could explain the complexity of observed effects induced by NikkomycinZ, such as differences in intensity and quality of effects (survival and malformation rates, mineral texture, shell solubility etc.) observed in larvae of various age. At the present stage it cannot be excluded that the chitin synthase acts in an indirect way by serving "simply" as a polymer matrix supplier for biomineralization. Even then it may have an important function for the mechanical integrity of the shell (Fig. 12) as well as for the maintenance of the composite material's properties. As shown here, the mineral becomes more soluble in case of chitin depletion (Fig. 10). This observation is consistent with the rapid disappearance of "dead shells" from the culture, or at least the decrease in shell size, as often observed even in living animals (Fig. 4, Fig. 11). The observed increase in crystallinity (Fig. 11) may have something to do with the interplay between chitin and the acidic mollusc shell proteins, which are suggested to stabilize the amorphous mineral fraction [16].

Our observations presented here may also contribute to an implementation of mechanical signal transduction into mollusc shell formation concepts. The mechanical functionality of the hinge gets lost whenever the formation of hinge teeth (see Fig. 9) is prevented. This might influence the formation of the valves as well, if one assumes a mechanical feedback mechanism that is responsible for the regulation of shell formation. If forces during valve closure are responsible for a correct valve formation, a direct correlation with mineral deposition should be guaranteed. An asymmetry of the valves (Fig. 4b) or an irregularly shaped shell edge (Fig. 5a,b, Fig. 6b,d), hinge line (Fig. 4a, Fig. 5c, Fig. 11e), or hinge teeth (Fig. 9d,h) will inevitably prevent a correct force application. Consequently, this mechanical feedback at the organism's level will interfere with stimulating the correct mechanically induced cell answer such as deposition of multiple organic and inorganic shell precursor components that are necessary for correct valve structuring. As the mollusc myosin chitin synthase strongly interferes with the actin cytoskeleton (unpublished results), multiple bottom-up effects are expected to be induced once the central coordination gets lost. Thus, the chitin synthase inhibitor NikkomycinZ has obviously the ability to interfere directly with one of the central players of this biomechanical coordination process.

Our continued research on larval shell biomineralization in *Mytilus galloprovincialis *aims to address the issue of chitin synthesis and structural modifications in the context of regulatory cascades that coordinate shell formation and the development of the shell forming tissue within the whole body plan *in vivo*.

## Conclusion

The small-molecule drug NikkomycinZ, a competitive chitin synthase inhibitor, was used at low concentrations (5–10 μM) for investigating the impact of chitin synthesis on mollusc larval shell formation *in vivo*. Being aware of the tremendous uncertainties that any *in vivo *approach bears, we observed dramatic structural alterations in mollusc larval shells by using polarized light video microscopy for the *in vivo *investigations and scanning electron microscopy imaging of extracted shell material prepared from NikkomycinZ treated *Mytilus galloprovincialis *larvae of various age. Provided that NikkomycinZ mainly affects chitin synthesis, our data obtained from different shell locations suggest that the mollusc chitin synthase fulfils an important enzymatic role in the coordinated formation of larval bivalve shells. It can be speculated that chitin synthesis bears the potential to contribute via signal transduction pathways to the implementation of hierarchical patterns into chitin mineral-composites such as prismatic, nacre, and crossed-lamellar shell types.

## Methods

### Mytilus galloprovincialis spawning and larvae culture

Larvae of *Mytilus galloprovincialis *were obtained after spontaneous spawning in the laboratory from February to April according to established protocols [61,62]. Artificial seawater was prepared from Reef Crystals™ (Aquarium Systems, Sarrebourg, France) and kept at a density of 1.022 g/cm^3 ^at 18°C. Adult animals of *Mytilus galloprovincialis *were obtained from Thaeron Fils (Riec, Belon, France). Animals were cleaned from algae, and thoroughly rinsed in tap water. Purified animals were put into a shallow tank containing 60 l of artificial seawater. After 2 h of recovery, 20 females were sorted out according to prespawning behavior and placed in a 40 l spawning tank. Animals were fed with 10 ml Aquatim™ phytoplankton (Kroon AQA^® ^/Z+L, Langen, Germany), and spawning males were added for 1–2 min in order to induce the females to spawn. The release of eggs occurred spontaneously during the following hours (see Additional File 1). 5 spawning male animals (see Additional File 2) were added for 30 min to complete fertilization. The fertilized eggs were harvested by using a combination of nylon membranes with 20 μm and 40 μm mesh size (see Additional File 3). The success of fertilization and the removal of potential contaminants were checked microscopically. The fertilized eggs were placed in 10 l of artificial seawater with slight aeration (see Additional File 4). The day of fertilization was defined as day zero (0-day). The developing embryos were fed with 5 ml Aquatim™ phytoplankton the following day. Every second day, the water was exchanged completely in order to remove contaminants, non-developing embryos, and dead larvae by increasing the nylon mesh sizes in intervals of ~20 μm according to the average size of healthy individuals. Mollusc larvae were fed with 5 ml Aquatim™ phytoplankton in several portions per day. Larvae of various developmental stages were obtained. About 22 to 29 days after fertilization, larvae reached the pediveliger stage with subsequent metamorphosis into the adult.

### Larvae culture in the presence of NikkomycinZ

Four series of experiments were performed, with each series covering selected developmental time frames of *Mytilus galloprovincialis *larvae. The time frames started 2, 5, 8, and 12 days after fertilization, and ended 5, 8, 12, and 15 days after fertilization, respectively. For each test culture, 100–200 Larvae were transferred from a 10l culture into 1 ml artificial seawater per well in a 12-well culture dish (Falcon, Heidelberg, Germany). NikkomycinZ (Sigma, Deisenhofen, Germany) was added in concentrations of 0 μM (control), 5 μM and 10 μM. Duplicate test cultures were performed. Every second day, the seawater was exchanged completely in order to remove contaminants and algal debris, and to avoid changes in salt concentration due to evaporation. NikkomycinZ was added freshly in the desired concentration for each test culture. Larvae were fed with 15 μl Aquatim^® ^(Z+L/Kroonaqa, Langen, Germany) phytoplankton once a day. The larvae cultures were incubated at 18°C with a 12 h light cycle.

A series of 40 ml scale cultures of 2-day, 5-day, 8-day, and 12-day *Mytilus galloprovincialis *larvae in the presence of 0 μM (control), 5 μM and 10 μM NikkomycinZ was performed for preparative purposes. The cultures were incubated at 18°C with a 12 h light cycle and aerated by gently shaking. Each culture was fed daily with 500 μl Aquatim^® ^phytoplankton. The seawater medium containing NikkomycinZ in the desired concentrations was exchanged every second day. Larvae were harvested after 3 days (earlier stages), and after about 10 days (later stages) of NikkomycinZ incubation on the 5^th^, 8^th^, 19^th^, and 22^nd ^day after fertilization, respectively, and processed immediately or stored shock-frozen in liquid N_2 _at -70°C.

### Evaluation of survival rate and phenotypes

Test cultures (1 ml scale) of *Mytilus galloprovincialis *larvae were frequently inspected using a stereomicroscope Wild M10 equipped with a Plan Apo 0,63× objective/10× zoom and a KL1500 electronic light source (Leica, Heerbrugg, Switzerland). The percentages of surviving and of abnormally developed larvae were estimated independently by up to three different persons with identical values within 10–20% deviation. The estimated deviation was thus defined as 20%.

### Video Microscopy

Larval phenotypes were investigated directly in the 12-well culture dish using video microscopy. An Axiovert 35 microscope (Zeiss, Oberkochen, Germany) equipped with LD ACHROPLAN 32×/0.40 Ph2 and LD ACHROPLAN 20×/0.40 Korr Ph2 objectives was used. Images were recorded with a colour video camera TK-1070BE (JVC, Friedberg, Germany) in S-VHS quality (AG-6730 Time lapse recorder, Panasonic, Hamburg) and digitized (Scenalyzer live: Andreas Winter, Nassau, Bahamas; TMPGEnc 3.0 × Press: Pegasys, Tokyo, Japan; and Premiere 7.0: Adobe Systems, München, Germany).

### Polarized Light Microscopy

Specimens were prepared on glass slides. Polarized light images were obtained by video microscopy as described in the previous section. The Axiovert 35 microscope (Zeiss, Oberkochen, Germany) was optionally equipped with crossed polarizers that were placed into the light path below and above the specimen. All images were taken by a constant light intensity of 11 arbitrary units.

### Scanning Electron Microscopy

For structural investigations of larval shells by scanning electron microscopy, shock frozen samples were thawed at room temperature prior to use. Larvae were washed 2× in deionised water. Larval shells were purified from tissue in 1% – 2.5% sodium hypochlorite for 7 minutes and washed in deionised water several times. Larval shells were placed in a small drop of deionised water on top of an ethanol cleaned specimen holder (stainless steel) and dried in air and under vacuum. Specimens were sputter coated with gold 3× for 30 seconds at 1,0 kV, 10 mA, and 3·10^-1^bar using a Scancoat Six Sputter Coater (Boc Edwards, Kirchheim, Germany). A Leo 1530 FESEM scanning electron microscope (Zeiss, Oberkochen, Germany) was operated at 2.0 kV and 6 mm working distance. The Leo-32 V03.00h software and Adobe Photoshop (Adobe München, Germany) was used for image processing.

## Competing interests

The author(s) declare that they have no competing interests.

## Authors' contributions

Both authors contributed equally to the experimental work. VS participated in the design of the study, and drafted the manuscript. IMW proposed the main idea, designed and coordinated the study, and drafted the manuscript. All authors read and approved the final manuscript.

## Supplementary Material

Additional File 1 **Video clip of spawning female**. Demonstration of spawning female and eggs prior to fertilization. Click here for file

Additional File 2 **Video clip of spawning male**. Demonstration of spawning male and sperm prior to fertilization. Click here for file

Additional File 3 **Video clip of fertilization procedure**. Demonstration of fertilization procedure and collection of fertilized eggs. Click here for file

Additional File 4 **Video clip of large scale larvae culture (10 l)**. Demonstration of the culture conditions of larvae in artificial seawater at 10 l-scale. Click here for file

Additional File 5 **Video microscopy of 2-day larvae**. Video microscopic (32× objective) demonstration of the behaviour of 2-day larvae. Note that the larvae are already in the early veliger stage and have a D-shaped shell. Click here for file

Additional File 6 **Video microscopy of 5-day larvae**. Video microscopic (32× objective) demonstration of the behaviour of 5-day larvae. The size of the velum and the motility of the larvae increased. Note that the hinge of the larval shell became straight. Click here for file

Additional File 7 **Video microscopy of 8-day larvae**. Video microscopic (32× objective) demonstration of the behaviour of 8-day larvae. The motility of the larvae is similar to 5-day larvae. The size of the larval shells slightly increased. Most of the shells are still in D-shape. Click here for file

Additional File 8 **Video microscopy of 12-day larvae**. Video microscopic (20× objective) demonstration of the behaviour of 12-day larvae. Note that the size of the shell increased, and the larvae switched from the D-shape stage to the umbo stage of shell formation. Click here for file

Additional File 9 **Video microscopy of 31-day larvae**. Video microscopic (20× objective) demonstration of the behaviour of 31-day larvae. Larvae are in different developmental stages, such as veliger larvae and pediveliger larvae. The latter are indicated by the developing foot and a degenerate, vanishing velum that is retracted into the shell. Click here for file

Additional File 10 **Video microscopy of 36-day larvae**. Video microscopic (20× objective) demonstration of the behaviour of 36-day larvae. At this age, the velum disappeared, and metamorphosis into the adult was completed as indicated by the functional foot. Click here for file

Additional File 11 **Table: Viability and malformation rate estimated in *Mytilus galloprovincialis *larvae populations in the presence of NikkomycinZ**. Data source (estimated values) of survival rate and malformation rate of mollusc larvae populations reared in the presence of 0 μM, 5 μM, and 10 μM NikkomycinZ as presented in figure 2 and figure 3. Click here for file

Additional File 12 **Video microscopy of 8-day larvae after 6 days of NikkomycinZ treatment**. Video microscopic (32× objective) demonstration of the behaviour and phenotype of 8-day larvae after 6 days of treatment with the chitin synthase inhibitor NikkomycinZ. The overall motility of the larvae decreased as compared to untreated control cultures. Note that NikkomycinZ appears to have a dramatic effect on shell development. The size of the shells is comparable to 2-day larvae. The hinge is not a straight line, and shell edges appear irregular or undulated. Click here for file

Additional File 13 **Video microscopy of 12-day larvae after 7 days of NikkomycinZ treatment**. Video microscopic (32× objective) demonstration of the behaviour and phenotype of 12-day larvae after 7 days of treatment with the chitin synthase inhibitor NikkomycinZ. The overall motility of the larvae decreased as compared to untreated control cultures. Note that NikkomycinZ appears to have a dramatic effect on shell development. The formation of the umbo was prevented (see additional file 8). The size of the shells is comparable to 5-day larvae. The most prominent features were curved hinges and malformed shell edges. Shells of some individuals were too small to host the organism completely. Click here for file

Additional File 14 **Video microscopy of 12-day larvae after 4 days of NikkomycinZ treatment**. Video microscopic (32× objective) demonstration of the behaviour and phenotype of 12-day larvae treated from the 8^th ^day on with the chitin synthase inhibitor NikkomycinZ. Even four days of treatment with NikkomycinZ have similar effects on shell development of individuals as described in additional file 13. Note that less affected individuals were highly motile and therefore out of focus in this data set. Click here for file

Additional File 15 **Video microscopy of 15-day larvae after 7 days of NikkomycinZ treatment**. Video microscopic (32× objective) demonstration of the behaviour and phenotype of 15-day larvae treated from the 8^th ^day on with the chitin synthase inhibitor NikkomycinZ. Even in later developmental stages, NikkomycinZ induced characteristic effects on the shell development of living individuals. Note that the previously straight hinge (see additional files 7 &8) appears now curved. This indicates that NikkomycinZ interferes with the remodelling of the hinge region. The fact that shells are much smaller than in the untreated control cultures (additional file 8) suggests that either the solubility of the newly formed shell is increased, or lateral shell growth is blocked by the chitin synthase inhibitor drug. Note that also shell remnants of larvae that died at undefined age are present in this data set. Click here for file
