# Supplementary material for: The structure of mollusc larval shells formed in the presence of the chitin synthase inhibitor Nikkomycin Z
Source: BMC Struct Biol. 2007 Nov 6;7:71. doi: 10.1186/1472-6807-7-71 (PMC2241824; doi:10.1186/1472-6807-7-71)
Supplement: Additional File 11 — Table: Viability and malformation rate estimated in Mytilus galloprovincialis larvae populations in the presence of NikkomycinZ. Data source (estimated values) of survival rate and malformation rate of mollusc larvae populations reared in the presence of 0 μM, 5 μM, and 10 μM NikkomycinZ as presented in figure 2 and figure 3. [file 1472-6807-7-71-S11.pdf]

Addition of NikkomycinZ after 2 days

Analysis of control

age (days)	survival rate (%)								mean	deviation
2	100	100	100	100	100	100	100	100	100	0
5	80	90	90	90	90	90	90	90	89	4
8	50	70	80	80	80	80	90	90	78	13
12	50	40	80	80	10	80	90	90	65	29
15	50	50	90	90	20	40	90	90	65	28

age (days)	malformation rate (%)								mean	deviation
2	0	0	10	10	20	20	0	0	8	9
5	10	10	10	10	20	20	10	10	13	5
8	40	40	20	20	50	50	10	10	30	17
12	90	90	30	30	20	20	20	20	40	31
15	80	80	10	10	20	20	20	20	33	30

Analysis of 5µM NikkomycinZ

age (days)	survival rate (%)								mean	deviation
2	100	100	100	100	100	100	100	100	100	0
5	30	40	50	50	80	80	60	70	58	18
8	10	10	20	10	30	0	20	20	15	9
12	0	0	10	10	0	0	0	0	3	5
15	0	0	0	0	0	0	0	0	0	0

age (days)	malformation rate (%)								mean	deviation
2	0	0	10	10	20	20	0	0	8	9
5	10	10	30	40	80	80	30	30	39	27
8	90	90	90	90	90	100	50	40	80	22
12	100	100	100	100	100	100	100	100	100	0
15	100	100	100	100	100	100	100	100	100	0

Analysis of 10 µM NikkomycinZ

age (days)	survival rate (%)								mean	deviation
2	100	100	100	100	100	100	100	100	100	0
5	30	30	50	10	10	20	50	60	33	19
8	10	10	10	0	30	30	20	40	19	14
12	0	0	0	0	0	0	0	0	0	0
15	0	0	0	0	0	0	0	0	0	0

age (days)	malformation rate (%)								mean	deviation
2	0	0	10	10	20	20	0	0	8	9
5	20	10	90	90	80	80	50	40	58	32
8	90	90	90	100	90	90	80	90	90	5
12	100	100	100	100	100	100	100	100	100	0
15	100	100	100	100	100	100	100	100	100	0

Addition of NikkomycinZ after 5 days

Analysis of control

age (days)	survival rate (%)								mean	deviation
5			100	100	100	100	80	80	93	10
8			80	80	90	90	10	10	60	39
12			90	90	60	80	10	10	57	38
15			90	90	20	30	10	30	45	36

age (days)	malformation rate (%)								mean	deviation
5			10	10	20	20	10	10	13	5
8			30	20	30	20	20	20	23	5
12			20	20	20	20	10	10	17	5
15			10	10	10	10	10	10	10	0

Analysis of 5µM NikkomycinZ

age (days)	survival rate (%)						mean	deviation
5	100	100	90	90	90	90	93	5
8	50	50	40	40	10	10	33	19
12	10	20	10	10	0	0	8	8
15	0	0	0	0	0	0	0	0

age (days)	malformation rate (%)						mean	deviation
5	10	10	20	20	10	10	13	5
8	70	60	80	90	40	50	65	19
12	100	100	90	100	100	100	98	4
15	100	100	100	100	100	100	100	0

Analysis of 10µM NikkomycinZ

age (days)	survival rate (%)						mean	deviation
5	100	100	80	80	90	90	90	9
8	20	0	40	40	10	20	22	16
12	10	0	10	0	0	10	5	5
15	0	0	0	0	0	0	0	0

age (days)	malformation rate (%)						mean	deviation
5	10	10	20	20	10	10	13	5
8	70	100	90	90	90	80	87	10
12	100	100	100	100	100	100	100	0
15	100	100	100	100	100	100	100	0

Addition of NikkomycinZ after 8 days

Analysis of control

age (days)	survival rate (%)								mean	deviation
8	100	100	100	100	100	100	100	100	100	0
12	40	50	90	90	90	90	90	90	79	21
15	30	50	90	90	40	40	90	90	65	27

age (days)	malformation rate (%)								mean	deviation
8	10	10	10	10	10	10	10	10	10	0
12	90	90	10	10	20	20	10	10	33	36
15	80	80	10	10	10	10	10	10	28	32

Analysis of 5µM NikkomycinZ

age (days)	survival rate (%)								mean	deviation
8	100	100	100	100	100	100	100	100	100	0
12	50	50	50	60	50	60	80	80	60	13
15	10	10	30	50	10	10	80	80	35	31

age (das)	malformation rate (%)								mean	deviation
8	10	10	10	10	10	10	10	10	10	0
12	90	90	10	10	50	50	20	20	43	33
15	100	100	10	10	10	10	20	10	34	41

Analysis of 10µM NikkomycinZ

age (days)	survival rate (%)								mean	deviation
8	100	100	100	100	100	100	100	100	100	0
12	10	10	80	80	80	80	90	90	65	34
15	10	10	50	50	10	10	70	70	35	28

age (days)	malformation rate (%)								mean	deviation
8	10	10	10	10	10	10	10	10	10	0
12	90	90	10	10	50	50	20	10	41	34
15	100	100	10	10	20	20	20	20	38	39

Addition of NikkomycinZ after 12 days

Analysis of control

age (days)	survival rate (%)								mean	deviation
12	100	100	100	100	100	100	100	100	100	0
15	70	70	90	100	90	70	90	90	84	12

age (days)	malformation rate (%)								mean	deviation
12	10	10	10	10	10	10	10	10	10	0
15	80	80	10	10	10	10	10	10	28	32

Analysis of 5µM NikkomycinZ

age(days)	survival rate (%)								mean	deviation
12	100	100	100	100	100	100	100	100	100	0
15	90	90	90	100	80	90	90	90	90	5

age (days)	malformation rate (%)								mean	deviation
12	10	10	10	10	10	10	10	10	10	0
15	90	90	10	10	10	10	10	10	30	37

Analysis of 10µM NikkomycinZ

age (days)	survival rate (%)								mean	deviation
12	100	100	100	100	100	100	100	100	100	0
15	90	90	90	80	50	50	90	90	79	18

age (days)	malformation rate (%)								mean	deviation
12	10	10	10	10	10	10	10	10	10	0
15	90	90	10	10	20	10	10	10	31	36
